# Society Meetings

**Published:** 1907-08

**Authors:** 


### SOCIETY MEETINGS.

### American Association of Obstetricians and Gynecologists.

THE American Association of Obstetricians and Gynecologists will hold its twentieth annual meeting at Detroit, Tuesday, Wednesday and Thursday, September 17, 18 and 19, 1907, under the presidency of Dr. Robert Tuttle Morris, of New York. Drs. J. S. Carstens, H. W. Longyear and W. P. Manton, all of Detroit, compose the committee of arrangements. An interesting program is in preparation. The following is a partial list of papers to be read:

1. The president's address, Robert Tuttle Morris, New York.
2. Intraabdominal torsion of the great omentum without hernia, R. E. Skeel, Cleveland.
3. Postoperative phlebitis, Daniel H. Craig, Boston.
4. Temporary uterovaginal fistula after panhysterectomy for fibroid of the uterus, Ernst Jonas, Saint Louis.
5. Puerperal sepsis, Charles E. Ruth, Keokuk.
6. Intestinal obstruction during pregnancy, Charles G. Cumston, Boston.
7. Some experiences relative to the causation and treatment of certain forms of metrorrhagia, Augustus P. Clarke, Cambridge.
8. Intestinal approximation or anastomosis by the use of the dumb bell, Joseph B. Bacon, Macomb, Ill.
9. Title to be announced, John B. Murphy, Chicago.
10. Title to be announced, Hugo O. Pantzer, Indianapolis.
11. The present treatment of puerperal sapremia and puerperal sepsis, J. E. Cannaday, Hansford, W. Va.
12. Deciduoma malignum, Miles F Porter, Fort Wayne.
13. Title to be announced, John D. S. Davis, Birmingham.
14. The causation of disease, with special reference to surgical diseases of the abdominal viscera, George E. Goodfellow, San Francisco.
15. A consideration of the toxemia of pregnancy as observed by the gynecologist, R. R. Huggins, Pittsburg.
16. Pelvic, abdominal, and constitutional subinvolution, S. W. Bandler, New York.
17. Title to be announced, A. Vander Veer, Albany.
18. Title to be announced, James E. Sadlier, Poughkeepsie.
19. Tuberculosis of the kidney, with report on operations in some advanced cases, Rufus B. Hall, Cincinnati.
20. Title to be announced, John B. Deaver, Philadelphia.

21. The formation of an artificial vagina by intestinal transplantation, J. F. Baldwin, Columbus.
22. Puerperal infection, Louis Frank, Louisville.
23. Title to be announced, Charles L. Bonifield, Cincinnati.
24. Title to be announced, J. H. Carstens, Detroit.
25. Title to be announced, Walter B. Dorsett, Saint Louis.
26. Title to be announced, George W. Crile, Cleveland.
27. Lithopedion; with a report of a case of 32 years standing, H. E. Hayd, Buffalo.
28. Typhoid perforation and its surgical treatment, Joseph Price, Philadelphia.
29. The relation of the weight of the placenta to the weight of the fetus, Walter P. Manton, Detroit.
30. Title to be announced, John Young Brown, Saint Louis.
31. Title to be announced, E. Gustav Zinke, Cincinnati.
32. The indications and contraindications for myomectomy and hysterectomy in uterine fibromyomata, James N. West, New York.
33. Cases illustrating common mistakes in gynecological diagnosis, William S. Smith, Baltimore.
34. Removal of large intraligamentary fibroid tumors without mutilation of any pelvic organ; two cases, Henry Howitt, Guelph.
35. Nephrocolopexy; report of cases, H. W. Longyear, Detroit.
36. The menstrual function; its influence upon chronic inflammatory conditions of the appendix, Francis Reder, Saint Louis.
37. Premature interruption of pregnancy, John A. Lyons, Chicago.
38. Title to be announced, Walter Wyman, Washington.
39. The treatment of endometritis and allied conditions of the uterus, Charles A. L. Reed, Cincinnati.

---

THE New York State Conference of Health Officers will hold its next annual meeting at Buffalo, October 14-19, 1907. Every health officer in the state is a member by law and must either attend in person or by substitute. It is estimated that at least 1,200 sanitary officers will be in attendance. It is announced that Governor Hughes will attend the opening meeting. A special feature of the conference will be the tuberculosis and hygenic exhibit at convention hall, which will constitute a museum of preventive medicine showing what is being done in this state for the prevention of disease. The meeting will be held under the auspices of the state department of health of which Dr. Eugene H. Porter is commissioner.

The Lake Keuka Medical and Surgical Association held its annual meeting at Grove Springs, N. Y., Thursday and Friday, July 25 and 26, 1907. An interesting program was dealt with.

The Eighth District Branch of the Medical Society of the State of New York will hold its annual meeting at Buffalo, Wednesday and Thursday, September 25 and 26, 1907. Members of the society who desire to read papers are requested to send titles thereof at an early day to Dr. Lee M. Francis, secretary, 482 Delaware Avenue, Buffalo.

